# What is the effectiveness of beta-glucan for treatment of acute otitis media?

**DOI:** 10.1016/j.bjorl.2020.02.004

**Published:** 2020-03-19

**Authors:** Erdem Atalay Cetinkaya, Osman Ciftci, Saadet Alan, Mustafa Namık Oztanır, Nese Basak

**Affiliations:** a University of Health Sciences, Antalya Training and Research Hospital, Department of Otorhinolaryngology and Head & Neck Surgery, Antalya, Turkey; b University of Pamukkale, Faculty of Medicine, Department of Pharmacology, Denizli, Turkey; c University of Inonu, Faculty Medicine, Department of Pathology, Malatya, Turkey; d University of Inonu, Faculty of Medicine, Department of Neurosurgery, Malatya, Turkey; e University of Inonu, Faculty of Pharmacy, Department of Pharmaceutical Toxicology, Malatya, Turkey

**Keywords:** Beta Glucan, Acute otitis media, Histological examination, Cytokines, Rats

## Abstract

**Introduction:**

As a supplement, beta–glucan has various therapeutic healing effects generated by the immune cells. It has been scientifically approved and proven to be a biological defense modifier. The aim of this study was to investigate the effects of beta–glucan on treatments administered in an acute otitis media model

**Objectives:**

This study investigated the effect of beta–glucan on the treatment of acute otitis media in an acute otitis media -induced animal model. Efficacy was evaluated both immunologically and histologically.

**Methods:**

The study sample comprised 35 adult rats, randomly separated into 5 groups of 7: Group 1 (control), Group 2 (acute otitis media, no treatment), Group 3 (acute otitis media + antibiotic), Group 4 (acute otitis media + beta–glucan) and Group 5 (acute otitis media + beta–glucan + antibiotic). Analyses were made of the histopathology and immunology examination results in respect of thickening of the tympanic membrane, epithelium damage, inflammation, and sclerosis. In all groups the serum levels of TNF-α, IL-4, IL-6 and IL-1β were evaluated.

**Results:**

All serum cytokine levels were significantly lower in the beta–glucan and antibiotic-treated groups compared to the acute otitis media Group. Significant differences in tympanic membrane thickness, inflammation, epithelium damage, and sclerosis values were observed between the acute otitis media + antibiotic and acute otitis media + beta–glucan Groups. According to these parameters, the values in aute otitis media + antibiotic + beta–glucan Group were markedly lower than those of the other groups. There was a significant difference in the acute otitis media + antibiotic + beta–glucan Groups compared to acute otitis media Group (*p* < 0.001).

**Conclusions:**

Both antibiotic and beta–glucan treatment reduced acute otitis media signs of inflammations in an acute otitis media-induced rat model, decreasing histological damage and cytokine levels. Co-administration of antibiotic and beta–glucan led to a significant reduction in tympanic membrane thickness, inflammation, and epithelium damage. Antibiotic + beta–glucan treatment resulted in a greater decrease in tympanic membrane thickness, inflammation, and epithelium damage than in the other groups. From these results, it can be suggested that beta–glucan, in combination with antibiotics may provide an alternative for the treatment of acute otitis media.

## Introduction

Acute otitis media (AOM) is seen extremely frequently during childhood and 75% of pre-school age children have been reported to have suffered at least one episode. Intracranial spread of infection is among the complications that may develop. Within the middle ear cavity (MEC), the mechanisms of AOM in the MEC which could make an individual susceptible to the development of inflammation are only partially known.^1^, ^2^ The 5 most common bacteria associated with AOM are *Streptococcus pneumoniae, Haemophilus influenzae, Moraxella catarrhalis, Streptococcus pyogenes,* and *Staphylococcus aureus*. The most common viruses that can contribute to AOM are human rhinovirus, influenza viruses, respiratory syncytial virus, adenovirus, and enterovirus. Patients with AOM suffer a reduced quality of life because of pain, hearing loss, headaches, and cognitive impairment.^3^, ^4^ A major contributing factor to AOM pathophysiology is the impaired function of the middle ear mucosa and subsequent stasis of infected secretions; this has been associated with local inflammation.^4^, ^5^

As AOM pathogens can be resistant to commonly-used antibiotics, proper treatment is challenging.^6^ Some AOM patients are prescribed supplementary and alternative therapies, particularly medicinal herbs, in addition to conventional treatments. Non-prescribed use of medicinal herbs for upper respiratory tract infection is common around the world. Beta-Glucan (β‒G) is a wide-spread plant constituent, which has been studied for decades in investigation of beneficial effects on humans. It has been scientifically approved and proven to be a biological defense modifier.^6^, ^7^ As a supplement, β‒G has various therapeutic healing effects generated by the immune cells. It can trigger development of a group of immune cells including macrophages, neutrophils, monocytes, natural killer cells, and dendritic cells, and it can inhibit tumor growth in the promotion stage.^7^, ^8^, ^9^ The aim of this study was to investigate the effects of β‒G on treatments administered in an experimentally-induced AOM rat model and to evaluate the results both immunologically and histologically.

## Methods

### Animals and experimentally-induced AOM

The groups were separated for treatment as follows: to Groups 2, 3 and 4, *Staphylococcus aureus* strain ATCC 25923 in sterile solution (0.5‒1 × 10^8^CFU/mL) was administered bilaterally using a dental needle (0.1 mL) via the transtympanic route. AOM developed within 48 h. Hyperemia of the tympanic membrane was observed macroscopically on day 2, and *S. aureus* growth was confirmed by culture. (Approval number of the ethics committee: 2015/A-56).

### Treatment groups

The 35 rats were randomly separated into 5 groups of 7. Group 1 (control) was administered 0.1 mL saline via the intratympanic route, followed by 0.01% Carboxymethyl Cellulose (CMC) via gavage. For Group 2 (AOM), 0.1 mL solution of *S. aureus* was administered via the intratympanic route to induce AOM, followed by 0.01% CMC via gavage. In Group 3 (AOM + antibiotic), following AOM induction, 50 mg/kg ampicillin was administered via gavage. In Group 4 (AOM+β‒G), following AOM induction, 50 mg/kg β‒G was administered via gavage. In Group 5 (AOM+β-G + antibiotic) following AOM induction, 50 mg/kg antibiotic and 50 mg/kg β‒G were administered via gavage. Ampicillin was administered twice a day for 14 days, and the β‒G was also administered for 14 days. On day 14, all the rats were weighed, then ketamine hydrochloride(75 mg/kg) and xylazine (8 mg/kg) anesthesia was applied and the animals were sacrificed under general anesthesia. Blood samples were syringed from the left ventricle for immunological analysis. After whole blood centrifugation (3,000×g, 20 min, at 4 °C) sera were obtained. Tissue and sera samples were stored at −45 °C until further analysis.

### Cytokine analysis

Cytokine production was determined with Enzyme-Linked Immunosorbent Assays (ELISAs) using commercial kits according to the manufacturers’ instructions. Interleukin-1beta (IL-1β) (cat no: EK0393) and IL-4 (cat no: EK0406), levels were measured using anti-rat ELISA kits from BosterBio (Pleasanton, CA, USA). IL-6 (cat no: KHC0061) and Tumor Necrosis Factor-alpha (TNF-α) (cat no: KRC3011) were obtained from Invitrogen (Carlsbad, CA, USA). Microtiter plates were read at 450 nm using the CA-2000 ELISA microplate reader (CIOM Medical Co., Ltd., Changchun, China). Using linear regression analysis, cytokine levels were calculated from standard curves of recombinant cytokines.

### Histopathological analysis

After sacrifice of the rats, the temporal bone was dissected from the skull. After opening the bullae, tissues were fixed in 10% buffered formalin, then incubated in 5% formic acid and routine tissue processing was performed for decalcification. Sections 4 μm in thickness were cut from the paraffin blocks and stained with hematoxylin and eosin (H&E). All the slides were examined under light microscopy (Olympus BX-51; Olympus, Tokyo, Japan) and images were captured using a digital DP70 camera attached to the microscope (Olympus). In the histological examination, evaluation was made of thickening of the tympanic membrane (ThicTM), damage to the epithelium (DamEpith), inflammation (Inf), and sclerosis (Sc). The severity of changes was scored as follows: none (-), mild (+), moderate (++), and severe (+++)^10^, ^11^ The score of none (-) was defined as normal epithelial and connective tissue; mild (+) as mild infiltration of individual inflammatory cells or their clusters, mild degeneration of epithelial cells, mild connective tissue fibroblastic cell proliferation; moderate (++) as moderate infiltration of inflammatory cells, focal epithelial loss and moderate connective tissue fibroblastic cell proliferation; and severe (+++) as dense infiltration of inflammatory cells and loss of epithelial integrity and marked connective tissue fibroblastic cell proliferation.

For statistical analysis these scores were equated to numerical scores (-)/0, mild (+)/+1, moderate (+ +)/+2, severe (+++)/+3. Quantitative assessment was made of the Thic TM thickness from measurement of the width of the tympanic membrane at the external edge facing the external auditory canal space and the width of the tympanic cavity facing the tympanic cavity at 10 different points.^11^ Epithelial damage was assessed by loss and erosion of the epithelium lining the tympanic membrane surface. Inflammatory cell increase was examined to evaluate inflammation. There was an increase in capillary vessels, congestion and edema. In the process of sclerosis; increased fibroblastic cells and myxoid degeneration were observed.

### Statistical analysis

Statistical analysis of the study data was made using SPSS for Windows software (ver. 18.0; SPSS Inc., Chicago, IL, USA). The results were stated as mean ± standard deviation (SD) values. The TNF-α, IL-4, IL-6, and IL-1β levels were compared between the treatment groups using One-way analysis of variance (ANOVA) and the post-hoc Duncan test. Kruskal-Wallis variance analysis was applied to the histological results. When differences were detected between the groups, the groups mean values were compared using the Mann-Whitney *U* test. A value of *p* < 0.01 was considered statistically significant.

## Results

The serum levels of TNF-α, IL-4, IL-6 and IL-1β for all the treatment groups are shown in Table 1. In the AOM Group, serum levels of TNF-α, IL-4, IL-6 and IL-1β increased significantly compared to the other groups (*p* <  0.01). The serum levels of TNF-α, IL-4, IL-6 and IL-1β were significantly decreased in the β‒G-treated and antibiotic-treated groups compared to the AOM Group. No significant differences were determined between the β‒G, antibiotic, and β-G+antibiotic-treated groups in respect of cytokine levels. The cytokine levels in these groups were observed to be similar to those of the control group.

**Table 1:** Levels of various cytokines in rats from different treatment groups (n = 7, mean ± SD).

	IL-1β	IL-4	IL-6	TNF-α
Control	10.5 ± 6.31^a^	22.3 ± 5.37^a^	27.6 ± 2.16^a^	5.61 ± 2.54^a^
AOM	38.3 ± 7.96^b^	41.7 ± 8.44^b^	38.2 ± 2.25^b^	11.5 ± 2.57^b^
AOM + antibiotic	14.5 ± 1.43^ac^	25.1 ± 6.83^a^	27.9 ± 3.12^a^	3.54 ± 2.43^a^
AOM+ β‒G	16.2 + 3.33^c^	27.7 ± 7.68^a^	32.0 ± 8.91^c^	5.01 ± 2.28^a^
AOM + antibiotic+ β‒G	16.8 ± 2.16^c^	26.9 ± 5.93^a^	33.6 ± 6.24^c^	4.95 ± 1.32^a^

^a,b,c^Different superscripts within the same column indicate statistically significant differences (*p* <  0.01).

SD, Standard Deviation; AOM, Acute Otitis Media; IL, Interleukin; TNF, Tumor Necrosis Factor; β‒G (Beta–Glucan).

### Histopathological results

In the histological examination, the control group was observed to have a clear MEC, external auditory Canal (eAC) and a thin, single cell mucosal lining of the cavity. In the AOM groups, normal EAC was observed, but there was effusion within the MEC and a thickened mucosa, with lamina propria including polymorphonuclear leukocytes, histiocytes, fibroblasts and granulation tissue. Cavity effusion in affected rats contained foamy macrophages and neutrophils.

**Fig. 1:**
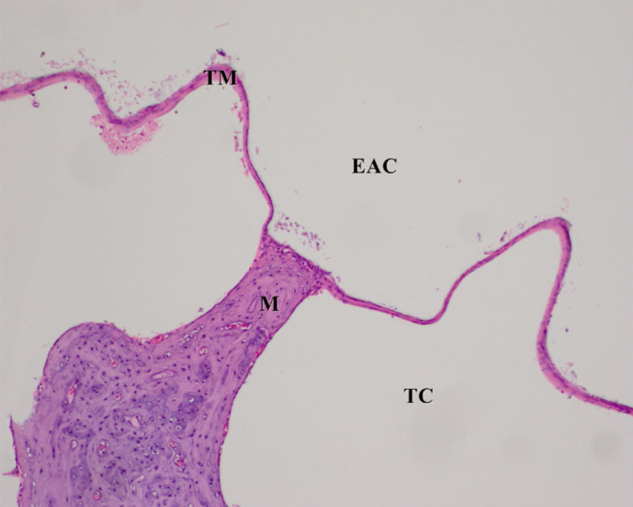
Control group, Tympanic membrane structure and malleus attached to membrane; H&E ×100. (EAC, External Auditory Canal; TC, Tympanic Cavity; TM, Tympanic Membrane; M, Malleus; H&E, Hematoxylin and Eosin).

**Fig. 2:**
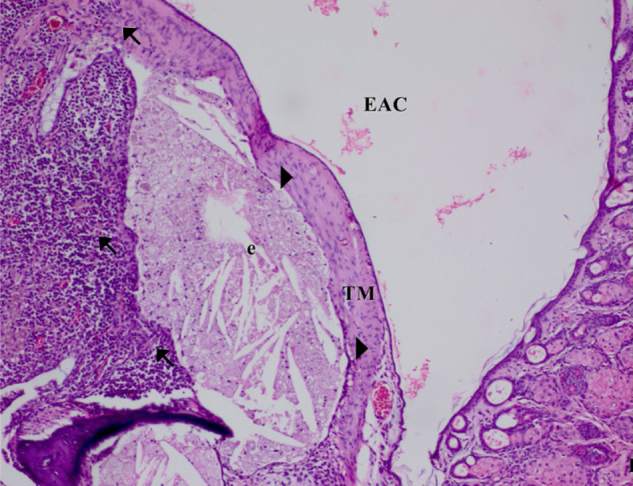
AOM Group. ThicTM, DamEpith, Inf, (arrow) of the connective tissue and sc (head arrow), exuda (e), ThicTM (score +3), DamEpith (score +3), Inf, and Sc (score +3). H&E ×100.

**Fig. 3:**
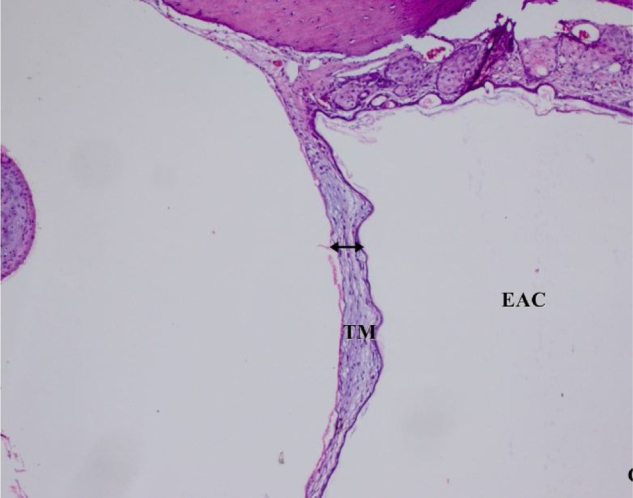
AOM + Antibiotic group. The marked decrease in ThicTM, DamEpith, Inf and Sc (head arrow), H&E ×100.

**Fig. 4:**
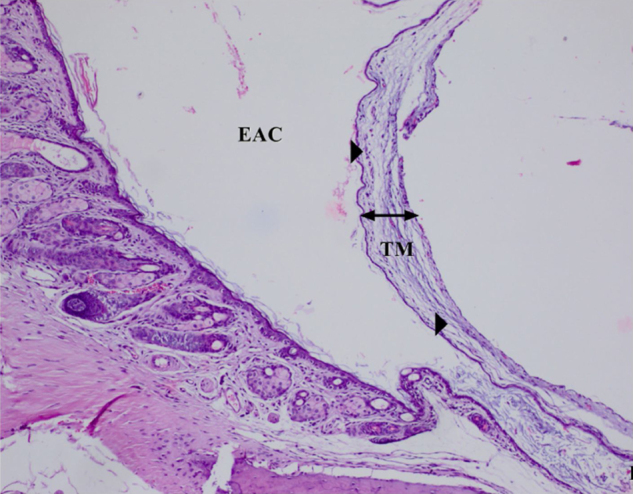
AOM+β‒G Group. The marked decrease in ThicTM, DamEpith and Inf (arrow), H&E ×100.

**Fig. 5:**
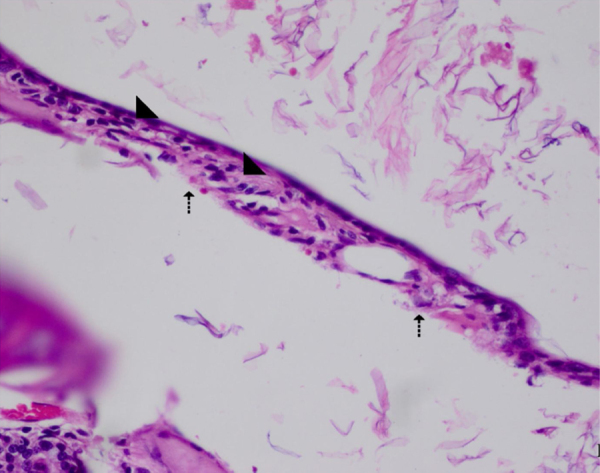
AOM + antibiotic+β‒G Group. The marked decrease in ThicTM, Inf and Sc (head arrow), DamEpith (cut arrow), H&E ×400.

The histopathological examination results of changes in the tympanic membrane in respect to thickening of the tympanic membrane (ThicTM), damage to the epithelium (DamEpith), inflammation (Inf), and sclerosis (Sc) are presented in Table 2 and Figs. 1–5.

**Table 2:** Histopathologic comparison of all groups according to tympanic membrane thickness, inflammation sclerosis and epithelial damage values.

	ThicTM	DamEpith	Inf	Sc
Control	15.25 ± 1.53^a^	0.00 ± 0.00^a^	0.00 ± 0.00^a^	0.00 ± 0.00^a^
AOM	100.35 ± 2.12^b^	2.50 ± 0.51^b^	2.83 ± 0.40^b^	1.60 ± 0.54^b^
AOM + antibiotic	24.41 ± 4.13^a^	0.60 ± 0.51^c^	0.60 ± 0.51^c^	0.50 ± 0.54^c^
AOM+β‒G	55.34 ± 4.29^c^	1.3 ± 0.70^d^	1.60 ± 0.51^d^	1.50 ± 0.44^b^
AOM + antibiotic+β‒G	46.25 ± 3.22^c^	0.50 ± 0.70 ^c^	0.50 ± 0.70^c^	0.50 ± 0.70^c^

^a,b,c^Different superscripts within the same column indicate statistically significant differences (*p* <  0.01).

ThicTM, Thickening of the Tympanic Membrane; DamEpith, Damage to the Epithelium; Inf, Inflammation; Sc, Sclerosis.

H&E stained sections demonstrated that the values of ThicTM, DamEpith, Inf, and Sc were significantly increased in the AOM group compared to the control group (Table 2 and Figs. 1‒2; *p* <  0.001).

The values of these parameters in the AOM + antibiotic group were markedly lower than those in the AOM Group (Table 2 and Figs. 2‒3; *p*< 0.001). In the AOM Group, ThicTM was increased with the migration of inflammatory cells and edema in the subepithelial layer of the bulla mucosa. The highest levels of DamEpith and Sc were determined in the AOM Group compared to all the other groups (Fig. 2).

No significant difference was determined between the AOM and AOM+ β‒G Groups in respect of the Sc values, and a significant difference was determined in respect of ThicTM, DamEpith and Inf values (Table 2, Figs. 2‒4; *p* < 0.001).

A significant difference was determined between the AOM + antibiotic and AOM+ β‒G groups in respect of ThicTM, Inf, and DamEpith values. No difference in Sc values was determined (Table 2, Figs. 3‒4).

The values of these parameters in the AOM + antibiotic+β‒G Group were lower than those of the other groups, with a significant difference in the values of the AOM + antibiotic+β‒G group compared to the AOM group (Table 2, Fig. 2, Fig. 3, Fig. 4, Fig. 5; *p* <  0.001).

Semi-quantitative evaluation was made of the changes observed. The arithmetic mean was obtained based on the AOM and control groups. In the control group, DamEpith, Inf, and Sc were not observed. An evident increase in ThicTM in the AOM group was observed due to inflammation and edema effect. Dense inflammation was caused by infiltration of “neutrophils and lymphohistiocytic cells” (2.83 ± 0.40). As a result of inflammation, degenerative changes and cellularity increase were observed in the connective tissue of the membrane wall. These changes occurred secondary to the inflammation in otitis media.

## Discussion

AOM is a common upper respiratory tract infection for which antibiotics are prescribed. It can affect all ages, with the greatest frequency reported in children. Moreover, AOM is the most common complaint associated with medical therapy for children < 5 years of age.^3^, ^4^

Antibiotic resistance has guided researchers to investigate new options. Medicinal herbs have been used for a long time, and in fact, continue to be used more often than prescribed medications.^8^, ^9^, ^10^ Current information proposes that these medicinal herbs exert a biological effect on immune regulation or cytotoxic mechanisms.^7^, ^8^, ^9^, ^10^, ^11^, ^12^ One of the immune-effective herbal products is in the form of complex polysaccharides known as β-glucans.^12^, ^13^ These biologically active polysaccharides found in both bacterial or fungal cell walls increase host immune defense by activating complementary systems and enhancing the functions of natural killer cells and macrophages. According to in vitro studies, β‒G interacts with several immune cell surface receptors, including Complement Receptor 3 (CR3; CD11b/CD18), dectin-1 (bGR), Toll-Like Receptors 2 and 6 (TLR-2/6), selected macrophage scavenger receptors and lactosylceramide. In addition, β‒G can inhibit tumor growth in the promotion stage.^8^, ^9^, ^12^, ^14^

In this study, the efficacy was evaluated of the use of a conventional antibiotic (ampicillin) and an herbal medication (β‒G), used in the management of anti-inflammatory and antimicrobial activities, in the treatment of AOM. The four histopathological examination parameters used to evaluate the effects of treatment were ThicTM, DamEpith, Inf, and Sc.^9^ It was seen that with the migration of inflammatory cells and edema into the subepithelial layer of the bulla mucosa, ThicTM was increased in the AOM Group. The highest DamEpith and Sc values were obtained in the AOM group. Compared to the AOM Group, all four values were significantly decreased in the AOM+β‒G + antibiotic group. According to these parameters, the values in the AOM+β‒G + antibiotic group were markedly lower than those of the other groups. It can be considered that this effect may be due to synergistic effects occurring through the combined use of the antibiotic and β‒G.

In previous experimental animal models, the role of cytokines in AOM has been well studied.^8^, ^9^, ^10^, ^11^ TNF-α, IL-1β IL-4 and IL-6 are known to be important local mediators associated with acute inflammation. For the evaluation of disease pathogenesis, the cytokine expression profile during AOM episodes is useful.^14^, ^15^, ^16^ In this study, the serum levels were measured of all four of these cytokines in the different treatment groups. The results showed that cytokine levels were significantly decreased in the β‒G-treated and antibiotic-treated groups compared to the AOM Group.

Several studies have revealed that β‒G regulates the production of various inflammatory cytokines. In addition, β‒G regulates host immune defence by activating the complementary system and enhancing the functions of natural killer cells and macrophages. These regulatory activities shed light on the potent effects of this herbal remedy.^8^, ^17^, ^18^ In addition to reducing the risk of (coronary) heart disease and anti-cancer actions, β‒G has also been reported to have a role in infection healing. β‒G has been approved as safe for us by the Food and Drug Administration. Furthermore, at all doses tested, no toxicity has been observed.^19^, ^20^, ^21^

In this prospective animal study, the effect of β‒G on the treatment of AOM was investigated in an AOM -induced rat model. Histopathological and immunological analyses were performed and the results were analyzed. To the best of our knowledge, there has been no previous study that has histologically and immunologically investigated the impact of β‒G on AOM.

## Conclusions

Animal model experiments related to otitis media have great benefits, such as subject manipulation and controlled disease/cure experimentation. The collective benefits of otitis media experimentation on animals has expanded the field for continuous and improved models on these subjectsFrom the results of this study, it can be suggested that β‒G, in combination with antibiotics, may provide a successful alternative treatment for AOM compared with antibiotics used alone. The favorable effects observed using β‒G are related to its immunomodulating activities. Therefore, the potential use of β‒G for the treatment of AOM should be further investigated. Future studies are required, which should focus on the use of β‒G in the treatment of AOM using randomized controlled studies in humans.

## Conflicts of interest

The authors declare no conflicts of interest.

## References

[1] Harker L.A., Shelton C., Cummings C.W., Flint P.W., Harker L.A., Haughey B.H., Richardson M.A., Robbins K.T. (2005). Otolaryngology-Head and Neck Surgery.

[2] Sidell D., Shapiro N.L., Bhattacharyya N. (2013). Obesity and the risk of chronic rhinosinusitis, allergic rhinitis, and acute otitis media in school-age children. Laryngoscope..

[3] Penido Nde O., Chandrasekhar S.S., Borin A., Maranhão A.S., Gurgel T. (2016). Complications of otitis media ‒ a potentially lethal problem still present. Braz J Otorhinolaryngol..

[4] Spektor Z., Pumarola F., Ismail K., Lanier B., Hussain I., Ansley J. (2017). Efficacy and safety of ciprofloxacin plus fluocinolone in otitis media with tympanostomy tubes in pediatric patients: a randomized clinical trial. JAMA Otolaryngol Head Neck Surg..

[5] Tapiainen T., Kujala T., Renko M., Koivunen P., Kontiokari T., Kristo A. (2014). Effect of antimicrobial treatment of acute otitis media on the daily disappearance of middle ear effusion: a placebo-controlled trial. JAMA Pediatr..

[6] Beta Glucan Research [Web site]. Available at http://www.betaglucan.org. Accessed Apr 8, 2019.

[7] Chan G.C., Chan W.K., Sze D.M. (2009). The effects of beta-glucan on human immune and cancer cells. J Hematol Oncol..

[8] Wasser S. (2002). Medical mushrooms as a source of antitumor and immunomodulating polysaccharides. Appl Microbiol Biotechnol..

[9] Birdane L., Muluk N.B., Cingi C., Burukoglu D., Fidan V., Incesulu A. (2014). Evaluation of the efficacy of curcumin in experimentally induced acute otitis media in rats. Ann Otol Rhinol Laryngol..

[10] Cetinkaya E.A., Ciftci O., Alan S., Oztanır M.N., Basak N. (2019). The efficacy of hesperidin for treatment of acute otitis media. Auris Nasus Larynx..

[11] Akramiene D., Kondrotas A., Didziapetriene J., Kevelaitis E. (2007). Effects of beta-glucans on the immune system. Medicina (Kaunas)..

[12] Shakeel M., Newton J.R., Ah-See K.W. (2009). Complementary and alternative medicine use among patients undergoing otolaryngologic surgery. J Otolaryngol Head Neck Surg..

[13] Barenkamp S.J., Kurono Y., Ogra P.L., Leiberman A., Bakaletz L.O., Murphy T.F. (2005). Recent advances in otitis media. 5. Microbiology and immunology. Ann Otol Rhinol Laryngol Suppl..

[14] Piltcher O.B., Swarts J.D., Magnuson K., Alper C.M., Doyle W.J., Hebda P.A. (2002). A rat model of otitis media with effusion caused by eustachian tube obstruction with and without Streptococcus pneumoniae infection: methods and disease course. Otolaryngol Head Neck Surg..

[15] Liu K., Kaur R., Almudevar A., Pichichero M.E. (2013). Higher serum levels of interleukin 10 occur at onset of acute otitis media caused by Streptococcus pneumoniae compared to Haemophilus influenzae and Moraxella catarrhalis. Laryngoscope..

[16] Li J.D., Hermansson A., Ryan A.F., Bakaletz L.O., Brown S.D., Cheeseman M.T. (2013). Panel 4: Recent advances in otitis media in molecular biology, biochemistry, genetics, and animal models. Otolaryngol Head Neck Surg..

[17] Ding J., Feng T., Ning Y., Li W., Wu Q., Qian K. (2015). β-Glucan enhances cytotoxic T lymphocyte responses by activation of human monocyte-derived dendritic cells via the PI3K/AKT pathway. Hum Immunol..

[18] Heinsbroek S.E., Williams D.L., Welting O., Meijer S.L., Gordon S., de Jonge W.J. (2015). Orally delivered β-glucans aggravate Dextran Sulfate Sodium (DSS)-induced intestinal inflammation. Nutr Res..

[19] Lee K.H., Park M., Ji K.Y., Lee H.Y., Jang J.H., Yoon I.J. (2014). Bacterial β-(1,3)-glucan prevents DSS-induced IBD by restoring the reduced population of regulatory T-cells. Immunobiology..

[20] Arena M.P., Caggianiello G., Fiocco D., Russo P., Torelli M., Spano G. (2014). Barley β-glucans-containing food enhances probiotic performances of beneficial bacteria. J Mol Sci..

[21] Topalsan S.U., Huseynov T., Sarıoglu S., Serbetcioglu B. (2012). Effects of penicillin and montelukast sodium on middle ear mucosa in rats with experimental acute otitis media. J Med Updates.

